# Distinct profiles of functional connectivity density aberrance in Alzheimer's disease and mild cognitive impairment

**DOI:** 10.3389/fpsyt.2022.1079149

**Published:** 2022-12-15

**Authors:** Dawei Miao, Xiaoguang Zhou, Xiaoyuan Wu, Chengdong Chen, Le Tian

**Affiliations:** ^1^School of Automation, Beijing University of Posts and Telecommunications, Beijing, China; ^2^School of Economics and Management, Minjiang University, Fuzhou, China; ^3^School of Electrical and Information Engineering, Beijing University of Civil Engineering and Architecture, Beijing, China

**Keywords:** Alzheimer's disease, mild cognitive impairment, fMRI, functional connectivity, default mode network

## Abstract

**Introduction:**

Investigating the neuroimaging changes from mild cognitive impairment (MCI) to Alzheimer's disease (AD) is of great significance. However, the details about the distinct functional characteristics of AD and MCI remain unknown.

**Methods:**

In this study, we investigated distinct profiles of functional connectivity density (FCD) differences between AD and MCI compared with the normal population, aiming to depict the progressive brain changes from MCI to AD. As a data-driven method, FCD measures the profiles of FC for the given voxel at different scales. Resting-state functional magnetic resonance imaging (fMRI) images were obtained from patients with AD and MCI and matched healthy controls (HCs). One-way ANCOVA was used to investigate (global, long-range, and local) FCD differences among the three groups followed by post-hoc analysis controlling age, sex, and head motion.

**Results:**

The three groups exhibited significant global FCD differences in the superior frontal gyrus. The post-hoc results further showed that patients with AD had a significant increase in global FCD values than those with MCI and HCs. Patients with MCI exhibited an increased trend compared with HCs. We further identified brain regions contributing to the observed global FCD differences by conducting seed-based FC analysis. We also identified that the observed global FCD differences were the additive effects of altered FC between the superior frontal gyrus and the posterior default model network.

**Discussion:**

These results depicted the global information communication capability impairment in AD and MCI providing a new insight into the progressive brain changes from MCI to AD.

## Introduction

As an irreversible neurodegenerative brain disorder, Alzheimer's disease (AD) leads to severe mental dysfunction and ultimately death in patients (1). AD is characterized by two main pathological changes, namely, amyloid-beta plaques and neurofibrillary tangles that finally lead to neuronal degeneration and loss (2). To date, there is no effective approach to early diagnosis and treatment that can stop or hinder this progression in the world (3). The identification of predictors at the beginning of AD, termed mild cognitive impairment (MCI) with an annual progression rate, from MCI to AD reaches 10 to 15% (4), which is of great significance in the clinic (5). Discovering potential biomarkers for identifying patients with MCI who are most likely to develop AD supports early diagnosis and medical intervention.

Advances in modern neuroimaging technologies, especially magnetic resonance imaging (MRI) technology, motivate researchers to identify distinct neuroimaging phenotypes between AD and MCI. Among these technologies, resting-state functional MRI turns out to be a powerful tool to investigate the progressive brain changes from MCI to AD (6). Studies recognized that both tau and amyloid-beta accumulation can affect neuronal activity and ultimately impair neuronal network (7, 8). Using the functional MRI, researchers consistently recognized that AD is accompanied by disruption of large-scale brain systems supporting a variety of cognitive abilities that were observed to decline with the disease progression (9). In other words, AD is a neurodegeneration featured with altered large-scale brain networks (10). These are two main methods to investigate intrinsic brain networks using resting-state functional MRI data, namely, independent component analysis (ICA) and seed-based functional connectivity (FC) (11). However, they have many problems. Specifically, seed-based approach relies on a predefined region of interest that is difficult to determine if the underlying pathology is unclear (12). As for ICA, there is no effective method to determine the appropriate number of independent components (13). In response to these problems, a novel method named FC density (FCD) mapping is proposed (14). As a data-driven method, FCD measures the number of functional connections between the given voxel and other voxels in the brain at different scales. Especially, the global FCD value is found to reflect the glucose metabolism (15) and the global information communication capability of the given voxel (16). Brain regions with high global FCD values are usually considered to be hubs of functional systems. At the same time, cascading network failure hypothesis postulates that tau deposition and amyloid lead to larger-scale brain network abnormalities, especially for functional hubs (9). In this regard, FCD is well suited to investigate brain disorders, including AD.

In this study, we aimed to investigate FCD differences among patients with AD and MCI and matched healthy controls (HCs) to depict the progressive brain changes from MCI to AD. Resting-state functional MRI images were obtained from patients with AD (*n* = 33) and MCI (*n* = 88) and HCs (*n* = 30). First, we calculated FCD (e.g., global FCD, long-range FCD, and local FCD) values for each subject. Then, we investigated FCD differences among the three groups. Previous studies found that MCI could be further divided into early MCI (EMCI) and late MCI (LMCI) on the basis of the severity of impaired delayed recall of logical memory (17). We also investigated whether EMCI and LMCI exhibited differences with regard to FCD. Finally, to further determine brain regions contributing to the observed FCD differences, seed-based FC maps were constructed where brain regions showing FCD aberrance were treated as seeds and compared among the three groups.

## Materials and methods

### Subjects

All subjects used in this study come from the Alzheimer's Disease Neuroimaging Initiative (ADNI) open database (http://adni.loni.usc.edu/). This project recruited more than 1,500 adults aged between 55 and 90 years since 2013, comprising patients with AD, those with MCI, and healthy population. We downloaded the dataset from ADNI phase 2. All subjects fulfilled the following inclusion criteria: (1) with no comorbidity with depression; (2) having no other kinds of dementia; (3) having clinical scales; and (4) having no obvious artifacts. In this dataset, cognitive function and degree of dementia were evaluated using the Mini-Mental State Examination (MMSE) and the Clinical Dementia Rating Scale-Sum of Boxes (CDR_SB) (18, 19). More details are included in Table 1.

**Table 1:** Demographic and clinical information of participants.

	**AD (*n* = 33)**	**MCI (*n* = 88)**	**HC (*n* = 30)**	***p*-Value**
Sex (female/male)	14/19	42/46	12/18	0.720^a^
Age (mean ± SE)	73.12 ± 7.36	71.60 ± 7.63	74.10 ± 5.60	0.219^b^
MMSE (mean ± SE)	20.65 ± 3.55	26.49 ± 2.13	28.93 ± 1.02	< 0.001^b^
CDR_SB (mean ± SE)	4.17 ± 1.68	1.65 ± 2.84	0.00 ± 0.00	< 0.001^b^

a Chi-square t-test.

b One-way ANOVA.

MMSE, Mini-Mental State Examination; CDR_SB, Clinical Dementia Rating Scale-Sum of Boxes.

Ethical review and approval were not required for the current study in accordance with the local legislation and institutional requirements. The datasets on which this article relies on were reviewed and approved by the Cleveland Clinic Institutional Review Board ADNI Individual Site Institutional Review Board. Written informed consent for participation was not required for this study in accordance with national legislation and institutional requirements.

### Data acquisition

Resting-state functional MRI images were acquired using a 3.0 T Philips Healthcare MRI scanner. All subjects were asked to keep their eyes closed during the scan. Images were obtained by echo-planar imaging (EPI) sequence. The scanning parameters were as follows: repetition time = 3 s, echo time = 30 ms, flip angle = 80°, acquisition matrix = 64 × 64, number of volumes = 140, slice thickness = 3.3 mm, and voxel size = 3 × 3 × 3 mm.

### Data preprocessing

The preprocessing of functional MRI images was performed using the Data Processing Assistant for Resting-State fMRI package (http://www.restfmri.net). The following steps were included: First, the first 10 scans with time point correction and realignment were removed. Then, images were normalized to the standard EPI template and resampled to 3 mm^3^. In this step, to control the head motion, subjects would be excluded if the translational/rotational displacement exceeded 3.0 mm/3.0°. Next, images were smoothed with 6 mm^3^ full-width at half maximum Gaussian kernel, detrended, and filtered with bandpass (0.01–0.1 Hz). Nuisance covariates including white matter signal, cerebrospinal fluid signal, and Friston 24 motion parameters (20) were regressed out. Especially, the global signal was not included as another covariate, as previous studies consistently recognized that the global signal bore physiological signification and was altered in mental disorders (21–23). Finally, to further remove the effect of head motion, scrubbing with cubic spline interpolation was used. The “bad” points were identified with a threshold of frame displacement larger than 0.5 mm as well as one-forward and two-back neighbors (24). The mean frame-wise displacement (FD) for each subject was also calculated (25, 26).

### Calculation of FCD

For each subject, we calculated (global, long-range, and local) FCD maps according to the previous study (14). In this study, we briefly described the calculation process, and more details could be referred to in the study of Tomasi and Volkow (14). For each voxel, the global FCD value was defined as the number of significant functional connections between it with other voxels in the gray matter. The local FCD value of one voxel was defined as the size of a continuous cluster of spatially connected voxels (voxel number) that were significantly correlated with it (27). The long-range FCD value of voxel *i* was obtained using the equation: long-range FCD_i_ = gFCD_i_ – local FCD_i_ (27). Some studies set the threshold with a predefined correlation *r* (e.g., 0.6). We did not adopt this strategy, as there was no clue to choose the optimal threshold (28). In this study, the significance of one connection was determined according to its *p*-value (*p* < 0.05, Bonferroni correction for all voxels in the gray matter) (29). Finally, all FCD maps were transformed to *Z*-scores by subtracting the mean and dividing the value by the standard deviation across gray matter voxels (30).

### Statistical analysis

We obtained (global, local, or long-range) FCD map differences among the three groups using one-way ANOVA equipped in SPM12 (http://www.fil.ion.ucl.ac.uk/spm). To exclude the effects of factors including age, sex, and mean FD, they were included as covariates in this step. The results were controlled with multiple comparisons with Gaussian random field (GRF) where voxel-wise threshold was a *p*-value of < 0.005 and cluster-level threshold was a *p*-value of < 0.05. To determine the details about between-group differences, we extracted the mean (global, local, or long-range) FCD values of each peak coordinate with a spherical radius of 6 mm that demonstrated significant differences among the three groups and compared them between each pair of groups with two-sample *t* test. As previous studies identified that MCI could be further divided into EMCI and LMCI (17), we also investigated whether FCD values (extracted before) exhibited significant differences between EMCI and LMCI with a two-sample *t* test.

### Identification of brain regions contributing to the FCD aberrance

To further identify which brain regions contributed to the observed FCD differences among the three groups, we calculated seed-based FC maps where peak coordinates of identified clusters showing FCD differences with a spherical radius of 6 mm were treated as seeds. The obtained FC values were transformed into Fisher *Z*-scores and then compared among the three groups followed by *post-hoc* analysis. This procedure was not designed to find significant FC differences connected to seeds but to identify brain regions contributing to the observed FCD differences. Thus, we reported uncorrected results with a loose threshold (voxel-wise *p* < 0.05, cluster size > 100).

### Association with symptom severity

To associate altered FCD values with symptom severity, Pearson's correlation coefficients between altered FCD values (extracted before) and symptom scores (CDR_SB/MMSE) were calculated.

### Head motion analysis

As head motion had a strong impact on FC, we adopted a number of strategies to exclude the effects of head motion on our results. First, subjects would be excluded if the translational and rotational displacement exceeded 3.0 mm or 3.0°. Second, mean FD was calculated for each subject and compared among the three groups. Third, we also calculated Pearson's correlation coefficients between the FCD values of brain regions exhibiting aberrance among the three groups and mean FD.

## Results

### Clinical demographics

The demographical and clinical information is included in Table 1. As we could see, the three groups demonstrated no significant differences in age and sex.

### ANOVA and post-hoc results of FCD

**Figure 1:**
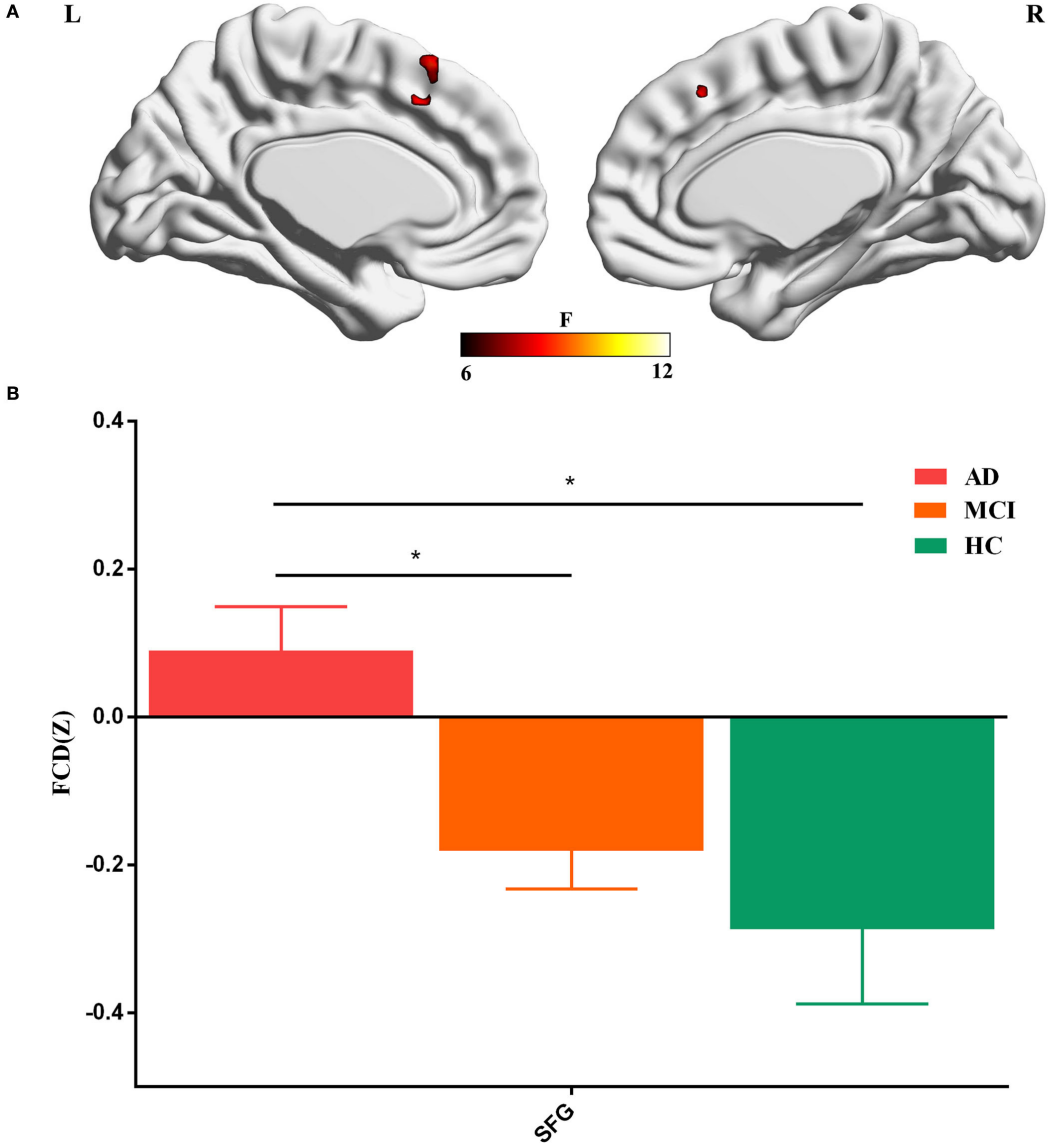
**(A)** Global FCD aberrance among the three groups. **(B)** *Post-hoc* results. SFG, superior frontal gyrus. The “*” means that the difference is significant (*p* < 0.05).

The three groups exhibited significant global FCD differences in the superior frontal gyrus (voxel-wise *p* < 0.005, cluster *p* < 0.05, GRF corrected; Figure 1, Table 2). The *post-hoc* results demonstrated that AD exhibited a significant increase of global FCD in the superior frontal gyrus than MCI (*t* = 2.948, *p* = 0.002, Cohen's *d* = 0.602) and HCs (*t* = 3.276, *p* = 0.004, Cohen's *d* =0.827). Patients with MCI exhibited an increased trend compared with HCs (*t* = 1.002, *p* = 0.318, Cohen's *d* = 0.212). There was no significant difference between patients with EMCI and LMCI (all *p* values > 0.05).

**Table 2:** Brain regions exhibiting global FCD aberrance among the three groups.

**Cluster**	**voxels**	**MNI (*x*, *y*, *z*)**	**Including regions**	***F***
1	75	9, 27, 48	Superior frontal gyrus	12.055
			Medial frontal gyrus	

### Brain regions contributing to the FCD aberrance

**Figure 2:**
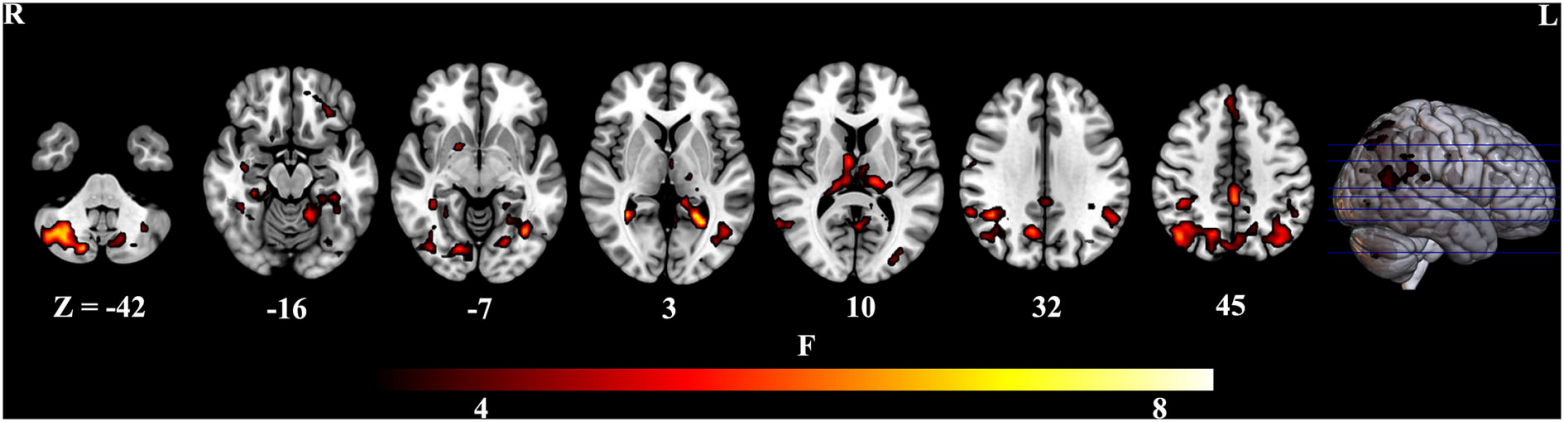
Brain regions contributing to altered global FCD.

**Figure 3:**
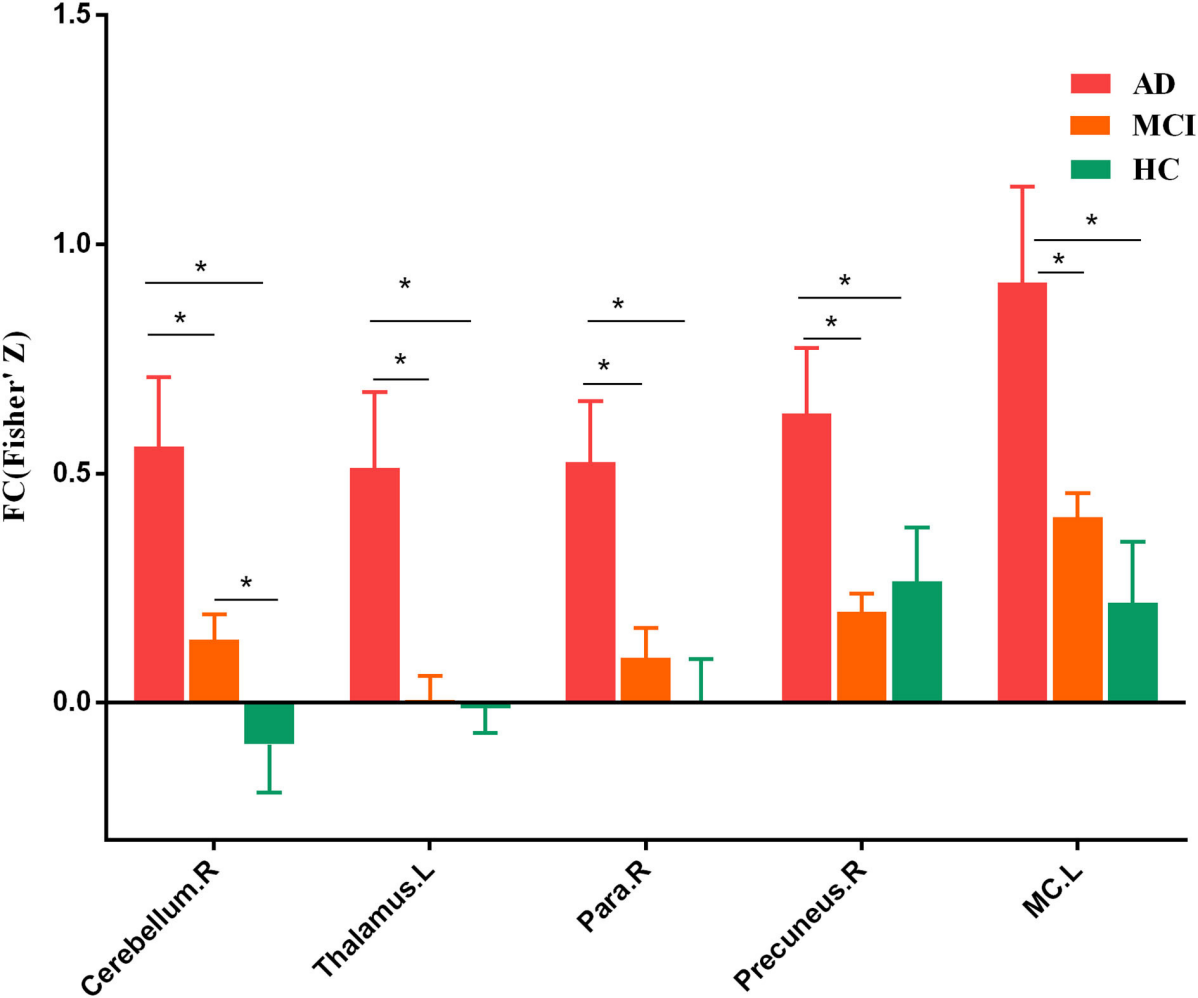
*Post-hoc* results of seed-based functional connectivity. Para, parahippocampus. MC, middle cingulum. The “*” means that the difference is significant (*p* < 0.05).

To further identify which brain regions contributed to the observed FCD differences, we constructed seed-based FC maps where the superior frontal gyrus was treated as the seed. The results demonstrated that distributed brain regions showed altered FC with the superior frontal gyrus, including the middle cingulum, the precuneus, the thalamus, the parahippocampus, the superior temporal gyrus, and the occipital lobe. The *post-hoc* results further showed that AD exhibited increased FC of these regions connected to the superior frontal gyrus than MCI and HCs (Figure 2). In addition, MCI exhibited increased FC linking the right cerebellum posterior lobe and the superior frontal gyrus than HCs. The *post-hoc* results are shown in Figure 3, Table 3.

**Table 3:** Brain regions contributing to the observed global FCD aberrance.

**Cluster**	**voxels**	**MNI (*x*, *y*, *z*)**	**Including regions**	***F***
1	566	30, −69, −42	Cerebellum posterior lobe	7.405
2	733	−36, −66, −3	Thalamus	9.461
			Parahippocampus gyrus	
			Occipital lobe	
3	188	33, −51, 3	Parahippocampus gyrus	7.794
			Fusiform gyrus	
4	1542	36, −36, 51	Precuneus	7.590
			Superior temporal gyrus	
			Middle temporal gyrus	
5	173	0, −36, 48	Middle cingulum	7.878
			Precuneus	

### Association with symptom severity

There was no significant correlation between global FCD values of the superior frontal gyrus and CDR_SB/MMSE scores (all *p*-values > 0.05).

### Head motion analysis results

We adopted a series of strategies to exclude the effects of head motion on our results. Four subjects (one AD, one MCI, and two HCs) were excluded if their translational and rotational displacement exceeded 3.0 mm or 3.0°. The three groups did not exhibit a significant difference in mean FD (*p* = 0.164). In addition, there was no significant correlation between mean FD and FCD values (all *p*-values > 0.05). These results indicated that our results were not obtained from head motion.

## Discussion

In this study, we investigated distinct profiles of FCD aberrance between AD and MCI, compared with matched HCs aiming to depict the progressive brain changes from MCI to AD. First, we found that the three groups exhibited a significant global FCD difference in the superior frontal gyrus. The *post-hoc* results further showed us that patients with AD had a significant increase in global FCD than those with MCI and HCs. Patients with MCI exhibited an increased trend compared with HCs. There was no significant difference between subtypes of MCI (EMCI and LMCI) in terms of global FCD values. We further identified brain regions contributing to observed global FCD aberrance. As a result, we identified that the observed global FCD aberrance was the additive effects of FC between the superior frontal gyrus and brain regions mainly located in the posterior DMN including the middle cingulum, the precuneus, the thalamus, the parahippocampus, the superior temporal gyrus, and the occipital lobe.

Implicated in a variety of cognitive processes and motor functions, the superior frontal gyrus was one of the brain regions showing the greatest age-related volume reduction and surface area reductions, which further predicts the risk of cognitive decline and dementia (31–33). Connected with distributed brain regions with white matter, the superior frontal gyrus was implicated in spatial working memory (34, 35). Lesion studies recognized that subjects with the superior frontal gyrus lesion exhibited impaired working memory performance, especially for spatial working memory (36). Decreased volume of the superior frontal gyrus was associated with disinhibited behavior in patients with AD (37). The intrinsic brain activity of the superior frontal gyrus was also found in AD (38). Coinciding with cascading network failure hypothesis, we observed increased global FCD values in AD, possibly reflecting a compensatory phenomenon in response to local network failure resulted from tau accumulation (9). The compensation mechanism was often reported and accompanied by impairments during the progression from MCI to AD (39–42). Patients with MCI also exhibited an increased trend of global FCD aberrance compared with HCs. These results suggested that the compensatory increase of the global information communication capability in the superior frontal gyrus might be related to the conversion from MCI to AD. Another possible explanation of the increased global FCD was the heterogeneity in the pathology of AD. Although functional dysfunction of the superior frontal gyrus was widely reported, the findings were conflicting (43, 44). This might have resulted from the high interindividual heterogeneity among individuals with AD. The high individual variation in etiology and clinical manifestations was increasingly acknowledged and was thought to be one of the leading causes resulting in conflicting findings in neuroimaging studies in brain disorders (45–49). In addition, we did not observe significant differences between EMCI and LMCI. A number of previous studies found that these two subtypes exhibited structural and functional aberrance differences (50–52). The disagreement between our results and previous studies might also be attributed to the heterogeneity. More future studies were needed to investigate the heterogeneity in AD and MCI.

Another finding was that the FC between the superior frontal gyrus and the brain regions mainly in the posterior default mode network (DMN) contributed to the observed global FCD aberrance. Although the pathology of AD was found to be related to a variety of brain networks, the dysfunction of DMN was the most consistent and frequent findings in AD (53). Compared with other brain networks, the DMN was preferentially studied for two main reasons. First, converging evidence recognized the linear association between the amyloid deposition and the dysfunction of DMN (54) whose core regions were associated with episodic memory retrieval (53, 55). Second, the dysfunction of DMN was related to the disease progression from MCI to AD (42, 56). For example, the hippocampus, playing a vital role in declarative memory, was identified as the anatomical signature of AD (57). The neuroanatomical aberrance of AD was thought to stem from the hippocampus and then spread to other brain regions (58). The hippocampal atrophy along with its atrophy rate was consistently found in AD and MCI and turned out to be potential biomarkers to forecast the conversion from MCI to AD (59, 60). Apart from brain regions in the DMN, the thalamus was found to play an important role in AD. The thalamus, receiving and integrating information from widespread brain regions, was of importance in cognitive processes, memory, and attention (61, 62). The microstructural change, volume atrophy, and functional decline led to a deficit in cognitive ability with age, as observed in the thalamus (63, 64). The volume atrophy and dysfunction of the thalamus were related to memory dysfunction in AD (65). Among these brain regions contributing to the observed global FCD aberrance, the cerebellum was noteworthy as its FC connected to the superior frontal gyrus differed between MCI and HCs. In addition to the motor function, recent evidence pointed out that the cerebellum was also implicated in working memory (66) and that working memory impairment was one of the dominating symptoms in AD and MCI (67). Consistent with these results, we found that FC between these brain regions and the superior frontal gyrus was altered in subjects with AD, helping to depict the progressive brain changes from MCI to AD.

This study has a number of limitations. First, all results were obtained in one single dataset, whether our conclusions held true in another independent dataset should be tested in the future. Second, longitudinal data declaring which subjects with MCI would develop AD were needed to further confirm our results. Third, another reason that we did not observe a significant difference between subtypes of MCI was the limited sample size. Future studies could confirm this by using datasets with large sample size.

## Conclusion

In this study, we investigated distinct profiles of FCD aberrance between AD and MCI compared with the normal population, aiming to depict the progressive brain changes from MCI to AD. Patients with AD exhibited a significant increase in global FCD than HCs/MCI and patients with MCI demonstrated an increased trend of global FCD compared with HCs. Further results identified that brain regions mainly located in the posterior DMN contributed to the observed global FCD aberrance. These results depicted the global information communication capability impairment in AD and MCI and provided a new insight into the progressive brain changes from MCI to AD.

## Data availability statement

The original contributions presented in the study are included in the article/supplementary material, further inquiries can be directed to the corresponding authors.

## Ethics statement

Ethical review and approval was not required for the study on human participants in accordance with the local legislation and institutional requirements. Written informed consent for participation was not required for this study in accordance with the national legislation and the institutional requirements.

## Author contributions

DM designed the research, analyzed the data, and wrote the manuscript. XW analyzed the data and wrote the manuscript. CC searched the literature and downloaded the data. LT provided suggestions and modified the language. XZ directed the research program and provided guidance and suggestions for the study. All authors read and approved the final manuscript.
